# Does hemolysis matter after pulsed field ablation for atrial fibrillation? Insights from the Best Ablate Registry

**DOI:** 10.1016/j.hroo.2025.11.010

**Published:** 2025-11-21

**Authors:** Alireza Sepehri Shamloo, Robert Hättasch, Claudius Sebastian Baldauf, Philipp Formum, Toshinori Chiba, Philipp Attanasio, Felix Hohendanner, Nikolaos Dagres, Gerhard Hindricks, Verena Tscholl

**Affiliations:** 1 Deutsches Herzzentrum der Charité, Klinik für Kardiologie, Angiologie und Intensivmedizin, Berlin, Germany; 2 Deutsches Herzzentrum der Charité, Klinik für Kardiologie, Angiologie und Intensivmedizin, Berlin, Germany

**Keywords:** Pulsed field ablation, Atrial fibrillation, Hemolysis, Renal failure, Troponin

## Abstract

**Background:**

Pulsed field ablation (PFA) has emerged as a novel nonthermal modality for atrial fibrillation (AF) ablation, offering tissue selectivity and procedural safety. However, emerging data have raised concerns about hemolysis-related biomarker shifts and potential renal failure after PFA.

**Objective:**

This study aimed to evaluate biomarker-defined hemolysis and the incidence of acute renal injury after PFA in patients undergoing AF.

**Methods:**

In this prospective observational study, 84 patients with paroxysmal or persistent AF undergoing PFA using either the Affera or PulseSelect system were enrolled. Paired venous blood samples were obtained before and 24 hours after the procedure. Biomarkers of hemolysis (lactate dehydrogenase [LDH], haptoglobin, hemoglobin, and bilirubin), renal function parameters (serum creatinine, urea, and electrolytes), and cardiac enzymes (high-sensitivity cardiac troponin, N-terminal pro-B-type natriuretic peptide) were systematically analyzed. Clinically significant hemolysis was defined as a postprocedural haptoglobin concentration of <0.04 g/L or a combination of indirect bilirubin of >1.5 times baseline and LDH more than twice baseline. Multivariable linear regression and mediation analyses were performed to identify procedural predictors of biomarker changes and explore the potential mediating role of myocardial injury.

**Results:**

Significant postprocedural changes were observed in biomarkers of hemolysis, including a reduction in haptoglobin (−0.17 ± 0.21 g/L; *P* < .001), an increase in LDH (+43.32 ± 79.98 U/L; *P* < .001), and bilirubin concentrations (*P* < .001). No patient met the predefined criteria for clinically significant hemolysis. Acute kidney injury stage 1, without oliguria, was observed in 3 patients (3.6%). Cardiac biomarker analysis revealed a substantial rise in high-sensitivity troponin (+1151.7 ± 750.8 ng/L; *P* < .001), whereas N-terminal pro-B-type natriuretic peptide decreased significantly after ablation (*P* < .001). The total number of PFA applications independently predicted changes in both LDH and haptoglobin.

**Conclusion:**

In our study, PFA for AF was associated with an increase in hemolytic parameters, without significant short-term renal impact or occurrence of clinically significant hemolysis. Hemolysis seems to be influenced by both lesion burden and myocardial injury, highlighting the need for further investigation into the long-term clinical relevance of biomarker-defined hemolysis in PFA-treated patients.

## Graphical abstract

**Figure:**
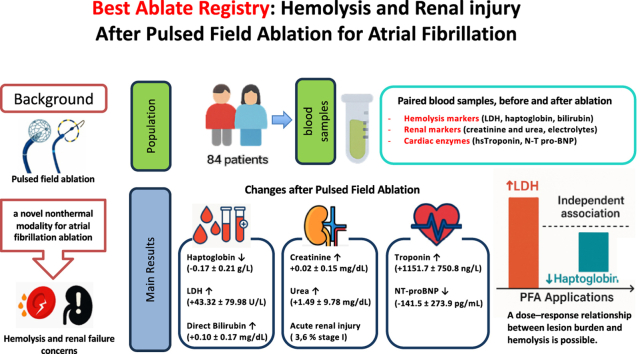

**Key Findings**

▪ Pulsed field ablation (PFA) for atrial fibrillation was associated with an increase in hemolytic parameters, without significant short-term renal impact or occurrence of clinically significant hemolysis.
▪ Acute kidney injury was rare (3.6%) and mild (Kidney Disease: Improving Global Outcomes stage 1 only), indicating that hemolysis after PFA does not usually translate into short-term renal dysfunction.
▪ The total number of PFA applications independently predicted hemolysis intensity, demonstrating a dose-dependent relationship between lesion burden and hemolytic biomarker change.

## Introduction

Pulsed field ablation (PFA) has emerged as a novel nonthermal energy technique for atrial fibrillation (AF) ablation.^1^^,^^2^ By applying high-voltage electric fields in the microsecond range, PFA induces irreversible electroporation of cardiomyocytes, achieving effective pulmonary vein isolation (PVI) with high tissue specificity.^3^ Early clinical studies have highlighted its safety advantages; nevertheless, some recent reports have raised concerns regarding intravascular hemolysis after PFA, which may contribute to acute kidney injury (AKI) mainly owing to hemoglobin-mediated nephrotoxicity.^4^, ^5^, ^6^

Although several studies have documented elevated hemolysis biomarkers and even AKI events in PFA-treated patients, systematic assessments using sensitive and specific markers remain scarce. Notably, both the extent of ablation and the number of PFA applications have emerged as potential risk factors for hemolysis and renal dysfunction.^7^^,^^8^ However, existing evidence is limited by heterogeneity in ablation protocols, inconsistent biomarker monitoring, and variable use of renal protective strategies such as hydration.^5^^,^^6^ Moreover, most hemolysis-related evidence has focused on the Farapulse PFA system, with limited data available on other PFA platforms such as the PulseSelect and Affera systems.

To address this uncertainty, we conducted this study to evaluate biomarker-defined hemolysis and its renal consequences after PFA for AF. We also aimed to assess the procedural predictors of hemolysis and explore whether cardiac injury mediates hemolysis-related effects.

## Methods

### Study design and patient population

This prospective observational study (Best Ablate Registry) was conducted at an academic center (Charité Campus Mitte, Berlin, Germany) in patients with paroxysmal or persistent AF who underwent PFA between February 2024 and July 2025.

Eligibility criteria included adult patients (age ≥18 years) undergoing their first or repeat catheter ablation for AF using PFA, with the ability to provide informed consent. Exclusion criteria were known hemolytic anemia, end-stage renal disease, or concurrent participation in another interventional trial.

### Study setting

All procedures and laboratory measurements were performed at a single academic tertiary care center (Charité – Universitätsmedizin Berlin), ensuring consistency in protocol and sample handling. The study was approved by the local ethics committee and conducted in accordance with the Declaration of Helsinki. All patients provided a written informed consent. This report adheres to the Strengthening the Reporting of Observational Studies in Epidemiology guidelines for observational studies.

### Procedural protocol

All patients were managed according to an uninterrupted oral anticoagulation protocol. In patients with uncertain adherence or subtherapeutic anticoagulation, transesophageal echocardiography was performed before ablation to exclude left atrial appendage thrombus. Procedures were conducted under either deep sedation with propofol and fentanyl or, if necessary, under general anesthesia. Systemic anticoagulation was achieved with intravenous heparin, titrated to maintain an activated clotting time of more than 300–350 seconds. Intravenous fluid administration was not protocolized and was left to the discretion of the operator.

### PFA

2 PFA systems were used: the Sphere-9 catheter with the Affera mapping and ablation system (Medtronic) and the PulseSelect PFA system (Medtronic). Each patient was assigned to 1 of the 2 PFA platforms according to institutional availability and operator discretion. After successful transseptal access, the Affera or PulseSelect system was deployed through its corresponding steerable sheath. In the Affera group, the Sphere-9 lattice-tip catheter was used with the Affera generator to deliver high-voltage pulsed field energy. In the PulseSelect group, pulsed energy was delivered through the PulseSelect catheter using bipolar biphasic waveforms. The number of applications per pulmonary vein and additional lesion sets (eg, posterior wall isolation) were performed based on operator preference and procedural end point criteria. Fluoroscopy and electroanatomic mapping were used to guide catheter positioning.

### Blood sampling

Venous blood samples were collected at 2 time points: (1) within 24 hours before the procedure and (2) 24 hours after ablation. Biomarkers related to hemolysis—including hemoglobin, haptoglobin, lactate dehydrogenase (LDH), bilirubin (total and direct), and reticulocyte count—were measured using standard clinical laboratory methods. Renal function was assessed through serial measurements of electrolytes, serum creatinine, urea, and estimated glomerular filtration rate. Cardiac biomarkers, including troponin and N-terminal pro-B-type natriuretic peptide (NT-proBNP), were also determined. Only patients with paired pre- and postprocedure samples were included in the final analysis.

### Procedural and technical metrics

The procedural protocol included detailed time stamping of events such as transseptal puncture, ablation initiation, and completion of PVI. Additional parameters analyzed included fluoroscopy time, radiation dose–area product, number of PFA applications per vein, and the total.

### Bias

To minimize selection bias, all consecutive eligible patients treated with either PFA system during the study period were included. No randomization or matching was used. To reduce measurement bias, standardized laboratory assays and imaging protocols were used.

### End points

The primary end points included changes in hemolysis-related biomarkers (LDH, haptoglobin, hemoglobin, and bilirubin), renal function markers and electrolytes, and cardiac enzymes. Significant hemolysis was defined as a postprocedural haptoglobin level of <0.04 g/L. In addition, a combination of indirect bilirubin of >1.5× baseline and LDH of >2× baseline was also considered indicative of significant hemolysis. AKI was defined according to the Kidney Disease: Improving Global Outcomes guidelines. Quantitative urine output was measured only in cases of suspected oliguria, defined as urine output of <0.5 mL/kg/h for ≥6 hours. Periprocedural complications, including thromboembolic, pericardial, and vascular access events, were also systematically recorded.

### Statistical analysis

Continuous variables were assessed for normality using the Shapiro–Wilk test and are presented as mean ± standard deviation or median with interquartile range, as appropriate. Categorical variables are reported as absolute counts and percentages. Paired comparisons of laboratory markers before and after PFA were performed using either paired *t* tests or Wilcoxon signed-rank tests, depending on data distribution. To explore the relationship between pre- and procedural parameters and hemolytic response, linear regression models were constructed using the absolute change (Δ) in selected hemolysis parameters as dependent variables. Key procedural parameters, including the total number of PFA applications, procedural duration, and fluoroscopy exposure, were included as covariates. Given the known rise in cardiac troponin after PFA, additional models were constructed to assess the potential confounding effect of myocardial injury. To further delineate whether troponin served as a mediator in the relationship between possible triggers and hemolysis, a stepwise mediation analysis was performed following the Baron and Kenny framework. 3 sequential linear regression models were calculated to estimate: (1) the association between total PFA applications and troponin change (path a), (2) the effect of troponin change on selected hemolysis parameters change (path b), and (3) the direct effect of total PFA applications on these parameters while adjusting for troponin (path c). The significance of the indirect effect (a × b) was tested using the Sobel method. Because of the limited number of AKI events observed in the cohort, formal predictive modeling was not performed for this outcome.

Sample size was determined pragmatically based on the number of eligible patients undergoing PFA during the predefined study period. No formal power calculation was performed. A 2-sided *P* < .05 was considered indicative of statistical significance. All statistical analyses were performed using SPSS (IBM SPSS Statistics for Windows, version 30.0), and supplementary procedures (eg, mediation analysis and Sobel testing) were performed using custom scripts in Python (v3.11).

## Results

### Baseline characteristics

During the study period, a total of 139 patients underwent PFA, of whom 84 (60.4%) had complete paired laboratory data and were included in the analysis. The mean age was 66.4 ± 9.0 years, with a slight male predominance (47 men and 37 women). The average body mass index was 26.4 ± 5.3 kg/m^2^. Of the study population, 60.7% (n = 51) underwent ablation with the Affera system, whereas 39.3% (n = 33) were treated using the PulseSelect system. Baseline cardiovascular risk profiles showed that 20.2% (n = 17) had known coronary artery disease, 10.7% (n = 9) had diabetes mellitus, 8.3% (n = 7) had a previous stroke or transient ischemic attack, and 58.3% (n = 49) had arterial hypertension. Mild to moderate mitral regurgitation was noted in nearly half of the patients (n = 40), and 5 patients (6.3%) had grade 3 severity. The mean left ventricular ejection fraction was 53.6 ± 8.7%, and the mean left atrial volume index was 45.9 ± 14.0 mL/m^2^. Most patients exhibited preserved renal function, with a mean estimated glomerular filtration rate of 85.6 ± 18.9 mL/min/1.73 m^2^. Chronic kidney disease stage 2 or 3 was observed in only a small subset of patients (n = 2 and n = 1, respectively), accounting for less than 5% of the cohort. The median CHA_2_D-VASc score was 2.0.

### Procedure-related characteristics

The mean total procedure duration was 88.0 ± 28.5 minutes, with a wide range between 35 and 163 minutes. On average, a total of 37.9 ± 26.1 PFA applications were delivered per patient across all pulmonary veins, with a mean of 19.4 ± 12.8 applications targeting the left pulmonary veins and 18.8 ± 15.3 targeting the right pulmonary veins. The mean fluoroscopy time was 742.6 ± 557.7 seconds, reflecting substantial variability across cases. The mean dose area product was 204.1 ± 181.9 μGy·m^2^.

### Use of analgesics and sedation

Sedation was performed using propofol, with a mean total dose of 764.3 ± 414.5 mg per procedure. All patients received analgesic medication during the procedure. The most administered analgesics were fentanyl (n = 81) and piritramide (n = 3).

### Changes in laboratory parameters before and after the procedure

#### Hemolysis-related markers

After PFA, a significant reduction was observed in hemoglobin (−0.93 ± 0.92 g/dL; *P* < .001) and reticulocyte counts (−4.14 ± 12.86 × 10^9^/L; *P* = .006). LDH levels increased significantly after the procedure, with a mean rise of +43.32 ± 79.98 U/L (*P* < .001). Both total and direct bilirubin concentrations increased significantly (total +0.30 ± 0.54 mg/dL; direct +0.10 ± 0.17 mg/dL; *P* < .001 for both). Haptoglobin levels showed a significant drop (−0.17 ± 0.21 g/L; *P* < .001) (Table 1). However, no cases fulfilled the predefined criteria for clinically significant hemolysis.

**Table 1:** Changes in hemolysis-related laboratory parameters before and after pulsed field ablation for atrial fibrillation

Parameter	Before the procedure (mean ± SD)	After the procedure (mean ± SD)	Change (mean ± SD)	Change direction	*P* value
Haptoglobin (g/L)	1.11 ± 0.54	0.94 ± 0.51	−0.17 ± 0.21	↓	<.001∗
LDH (U/L)	237.73 ± 66.47	281.27 ± 57.51	+43.32 ± 79.98	↑	<.001†
Hemoglobin (g/dL)	14.23 ± 1.73	13.31 ± 1.63	−0.93 ± 0.92	↓	<.001∗
Reticulocytes (×/nL)	74.90 ± 25.34	70.76 ± 19.52	−4.14 ± 12.86	↓	.006†
Total bilirubin (mg/dL)	0.69 ± 0.37	0.99 ± 0.68	+0.30 ± 0.54	↑	<.001∗
Direct bilirubin (mg/dL)	0.25 ± 0.11	0.34 ± 0.20	+0.10 ± 0.17	↑	<.001∗

LDH = lactate dehydrogenase; SD = standard deviation.

∗ Paired *t* test.

† Wilcoxon signed-rank test.

#### Renal function and electrolytes

No significant changes were observed in serum creatinine (+0.02 ± 0.15 mg/dL; *P* = .115) or urea levels (+1.49 ± 9.78 mg/dL; *P* = .304). However, a statistically significant decrease in serum sodium and potassium levels was observed (*P* < .001 for both) (Table 2). In 3 patients (3.6%), a postprocedural AKI stage 1 without oliguria was observed.

**Table 2:** Changes in renal function and electrolyte parameters before and after pulsed field ablation for atrial fibrillation

Parameter	Before the procedure (mean ± SD)	After the procedure (mean ± SD)	Change (mean ± SD)	Change direction	*P* value
Creatinine (mg/dL)	0.95 ± 0.27	0.97 ± 0.31	+0.02 ± 0.15	↑	.115∗
Urea (mg/dL)	34.65 ± 10.84	36.15 ± 13.21	+1.49 ± 9.78	↑	.304∗
Sodium (mmol/L)	138.86 ± 2.24	137.81 ± 2.58	−1.04 ± 2.17	↓	<.001†
Potassium (mmol/L)	4.32 ± 0.36	4.17 ± 0.39	−0.15 ± 0.41	↓	<.001∗

SD = standard deviation.

∗ Wilcoxon signed-rank test.

† Paired *t* test.

#### Cardiac enzymes

A significant increase in serum troponin levels was observed after the procedure, with (+1151.7 ± 750.8 ng/L; *P* < .001). In contrast, NT-proBNP levels demonstrated a statistically significant reduction after the procedure (−141.5 ± 273.9 pg/mL; *P* < .001) (Table 3).

**Table 3:** Changes in cardiac markers before and after pulsed field ablation for atrial fibrillation

Parameter	Before the procedure (mean ± SD)	After the procedure (mean ± SD)	Change (mean ± SD)	Change direction	*P* value∗
Troponin (ng/L)	38.0 ± 117.0	1189.7 ± 745.9 ng/L	+1151.7 ± 750.8	↑	<.001
NT-proBNP (pg/mL)	467.9 ± 386.0	326.3 ± 261.8	−141.5 ± 273.9	↓	<.001

NT-proBNP = N-terminal pro-B-type natriuretic peptide; SD = standard deviation.

∗ The Wilcoxon signed-rank test was used.

### Multiple linear regression

#### Predictors of haptoglobin change

In multivariable linear regression, the total number of PFA applications was independently associated with LDH elevation (β = 0.349; *P* = .035) and a significant reduction in haptoglobin levels (β = −0.417; *P* = .007), suggesting a dose–response relationship between lesion burden and hemolysis. No significant associations were observed with baseline patient characteristics or other procedure-related parameters. In contrast to LDH and haptoglobin, changes in hemoglobin levels were not significantly associated with baseline or procedural-related parameters, including the number of PFA applications (β = −0.116; *P* = .448).

#### Mediation analysis of troponin on hemolysis biomarkers

We performed a mediation analysis to assess whether the postprocedural rise in cardiac troponin mediated the relationship between the number of PFA applications and selected postprocedural hemolysis markers. The number of PFA applications was significantly associated with a rise in troponin levels (β = 15.33; *P* < .001). In turn, troponin increase was significantly associated with LDH elevation (β = 0.036; *P* = .028). However, when both predictors were entered simultaneously into the model, the direct association between the number of PFA applications and LDH changes became nonsignificant (β = 0.333; *P* = .496), suggesting a full mediation effect through troponin.

**Figure 1:**
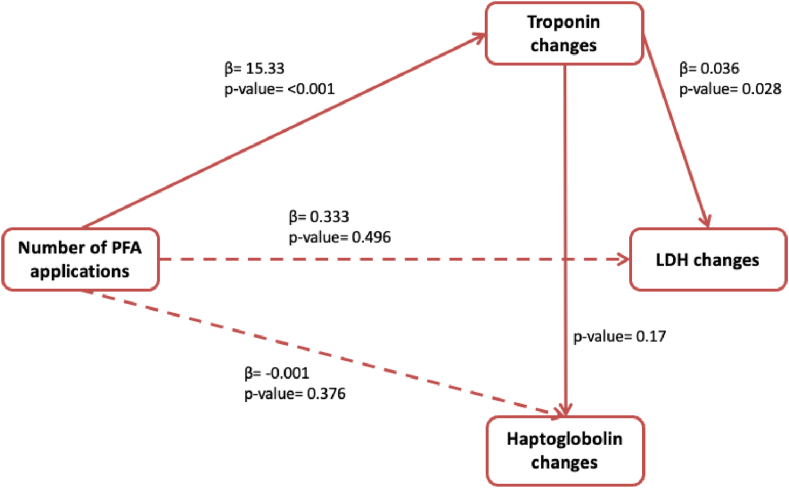
A visual model of the mediation analysis evaluating whether troponin rise mediated the relationship between PFA (number of applications) and selected hemolysis biomarkers (LDH and haptoglobin). LDH = lactate dehydrogenase; PFA = pulsed field ablation.

In contrast, no significant mediation effect was observed for haptoglobin. Although the number of PFA applications remained weakly related to haptoglobin changes (β = −0.001; *P* = .376), the path from troponin to haptoglobin changes was not statistically significant (*P* = .170), suggesting that troponin does not significantly mediate changes in haptoglobin after ablation. These findings indicate that although troponin mediates postablation LDH changes, the observed haptoglobin changes may not be driven by myocardial injury but possibly reflect pure hemolysis (Figure 1).

### Complications

Within 48 hours after PFA, 1 patient experienced a transient ischemic attack (1.2%). No cases of pericardial tamponade were observed. Vascular access complications occurred in 2 patients (2.4%) in the form of femoral vein pseudoaneurysms, both of which were successfully managed with manual compression, without the need for further intervention.

## Discussion

### Hemolysis after PFA: Prevalence, predictors, and mechanisms

In our prospective cohort of patients undergoing PFA for AF, we observed consistent biochemical changes indicative of hemolysis, including reductions in haptoglobin and elevations in LDH and bilirubin. These findings are aligned with previous studies investigating the hematologic impact of PFA.^4^^,^^8^^,^^9^ However, our study uniquely applied predefined, biomarker-based thresholds to define “clinically significant” hemolysis, and none of the patients in our cohort met these criteria. This approach, based on the work of Venier et al,^4^ required either a drop in haptoglobin of <0.04 g/L or a combined increase in LDH and indirect bilirubin, offering a different method compared with previous studies that relied on qualitative assessments or less stringent criteria. Previous studies, such as those by Popa et al^8^ and Stojadinović et al,^9^ reported hemolysis incidences as high as 28%–95%, but definitions varied substantially—often including elevations in free hemoglobin or isolated surrogate markers. Our study did not assess free hemoglobin or microparticles, which may partly explain the lower incidence observed. Disparities in biomarker timing, assay sensitivity, and procedural variables further complicate cross-study comparisons.^6^

1 key finding of our study was the independent association between the total number of PFA applications and the magnitude of hemolysis marker changes. Multivariable modeling confirmed this procedural metric as the strongest predictor of both LDH elevation and haptoglobin depletion. These findings support previous observations indicating a dose-dependent effect of lesion burden on hemolysis, with several studies identifying application thresholds—ranging from 54 to 74—beyond which hemolytic risk increases substantially.^4^^,^^8^

Importantly, our mediation analysis revealed that LDH elevations were also mediated by troponin release, suggesting that myocardial injury contributes significantly to LDH changes after PFA. In contrast, haptoglobin changes were not mediated by troponin, implying that red blood cell lysis occurs independently of cardiac damage. These results highlight the overlapping but distinct contributions of myocardial injury and hemolysis to the observed biomarker profiles.

Whether catheter design or energy delivery system influences hemolysis remains an open question. Although our study included both the Affera and PulseSelect systems, the sample size did not allow for formal system-level comparisons. Nevertheless, the presence of hemolytic biomarker changes across both platforms suggests that this phenomenon is not necessarily restricted to a specific device. Previous studies have suggested that large-profile catheters with wide electric field dispersion may increase red cell exposure to electroporation, particularly during high-output or prolonged applications.^7^^,^^10^

### Renal consequences: AKI risk and subclinical dysfunction

Despite biochemical evidence of hemolysis, AKI was rare in our cohort, occurring in only 3.6% of patients. These cases were limited to Kidney Disease: Improving Global Outcomes stage 1 and were not associated with oliguria or dialysis requirement. This observation is consistent with previous studies reporting low AKI rates after PFA.^4^^,^^8^^,^^11^ On average, serum creatinine and urea remained unchanged after the procedure, aligning with findings from prospective investigations such as that by Heiden et al^12^ and registry data from MANIFEST-17K.^11^

Nevertheless, it remains possible that kidney injury may occur beyond the immediate postprocedural window. Our study’s follow-up was restricted to 24 hours, which may not capture delayed renal effects. Moreover, the absence of significant hemolytic biomarker elevation in the few patients who developed AKI suggests that alternative mechanisms—such as hemodynamic fluctuations or subclinical tubular stress—may contribute. Unfortunately, the sample size precluded a formal risk factor analysis for AKI, and sensitive markers of early tubular injury were not assessed.

Previous work has shown that periprocedural hydration may mitigate the risk of PFA-associated renal injury.^13^ Mohanty et al,^5^ in a prospective study, reported complete AKI prevention in patients who received standardized hydration compared with a 14% AKI incidence in the control group. Although hydration protocols were not standardized in our study, this strategy seems promising and warrants incorporation into routine practice, particularly for patients with preexisting renal impairment.

### Cardiac injury: A potential mediator

Consistent with earlier findings, we observed a substantial increase in cardiac troponin levels after PFA, reflecting the expected myocardial injury from lesion creation.^14^ This elevation occurred despite the absence of procedural complications or structural sequelae such as pericardial effusion. Interestingly, NT-proBNP levels declined significantly after ablation. This counterintuitive finding may reflect acute reductions in atrial pressure or neurohumoral modulation after PVI.^15^ Comparative data on NT-proBNP dynamics after PFA are sparse, and future studies should evaluate whether this biomarker may aid in differentiating between benign and clinically relevant cardiac stress.

The overlap between hemolysis and cardiac injury becomes particularly relevant when interpreting LDH changes. Although LDH is a nonspecific marker traditionally associated with hemolysis, our mediation analysis supports a significant contribution from myocardial injury to LDH elevations. In contrast, haptoglobin—an acute-phase reactant and more specific marker of intravascular hemolysis—was not affected by troponin levels, reinforcing its utility for distinguishing hemolytic from cardiac injury. Our findings suggest that LDH may be interpreted cautiously in the context of PFA and that concurrent cardiac biomarkers are essential for accurate attribution. This distinction has important implications for clinical decision making and risk stratification after ablation procedures.

### Limitations

Several limitations should be acknowledged. The single-center design and the fact that highly experienced operators performed procedures may restrict the applicability of the findings to broader practice. Although prospective data collection and standardized sampling protocols strengthen internal validity, the observational nature of the study precludes causal inference. Follow-up of biomarkers was limited to the first 24 hours after the procedure, which means that later hemolytic or renal complications could have been missed. We also did not include more sensitive biomarkers (such as NGAL, KIM-1, or microparticles), which may have led to an underestimation of subtle injury. Furthermore, periprocedural hydration was not standardized or quantitatively documented, which may have influenced renal biomarker dynamics and should be systematically addressed in future studies. Finally, although the sample size is larger than in several earlier reports, it was still too small to support predictive modeling or detailed subgroup analyses, particularly for infrequent outcomes such as AKI. Comparative analyses between the 2 PFA systems were beyond the scope of this study but should be addressed in future work.

## Conclusion

In this prospective, biomarker-guided study, PFA for AF was associated with consistent biochemical evidence of hemolysis. Although haptoglobin and LDH levels changed significantly after ablation, no patient met the criteria for clinically significant hemolysis or developed severe renal complications. The number of PFA applications emerged as a consistent predictor of hemolysis-related biomarker changes. Troponin-mediated myocardial injury contributed to LDH elevation but did not explain reductions in haptoglobin, underscoring the intertwined nature of myocardial and hemolytic injury in this setting. These findings support the safety of PFA with current ablation strategies but emphasize the importance of application moderation, postprocedural monitoring, and renal protection strategies in future practice and research.

## Disclosures

V.T. reports receiving speaker honoraria from Medtronic, unrelated to the present work. All other authors declare no conflicts of interest.

## References

[1] Chen S., Narayan S.M., Boveda S. (2025). International expert practical guide on the use of the pentaspline pulsed field ablation system in atrial fibrillation ablation procedures. Circ Arrhythm Electrophysiol.

[2] Tzeis S., Gerstenfeld E.P., Kalman J. (2024). 2024 European Heart Rhythm Association/Heart Rhythm Society/Asia Pacific Heart Rhythm Society/Latin American Heart Rhythm Society expert consensus statement on catheter and surgical ablation of atrial fibrillation. Europace.

[3] Tabaja C., Mdaihly M., Noujaim C., Wazni O.M., Santangeli P. (2025). Tissue selectivity of pulsed field ablation. Card Electrophysiol Clin.

[4] Venier S., Vaxelaire N., Jacon P. (2023). Severe acute kidney injury related to haemolysis after pulsed field ablation for atrial fibrillation. Europace.

[5] Mohanty S., Casella M., Compagnucci P. (2024). Acute kidney injury resulting from hemoglobinuria after pulsed-field ablation in atrial fibrillation: is it preventable?. JACC Clin Electrophysiol.

[6] Xu Y., Gulburak T.K., Lu Y. (2025). Hemolysis after pulsed-field ablation of atrial fibrillation. Heart Rhythm.

[7] Nies M., Koruth J.S., Mlček M. (2024). Hemolysis after pulsed field ablation: impact of lesion number and catheter-tissue contact. Circ Arrhythm Electrophysiol.

[8] Popa M.A., Venier S., Menè R. (2024). Characterization and clinical significance of hemolysis after pulsed field ablation for atrial fibrillation: results of a multicenter analysis. Circ Arrhythm Electrophysiol.

[9] Stojadinović P., Ventrella N., Alfredová H. (2024). Prediction of major intravascular hemolysis during pulsed electric field ablation of atrial fibrillation using a pentaspline catheter. J Cardiovasc Electrophysiol.

[10] Boersma L.V.A., Széplaki G., Dello Russo A. (2025). Real-world experience with the pentaspline pulsed field ablation system: one-year outcomes of the FARADISE registry. Europace.

[11] Ekanem E., Neuzil P., Reichlin T. (2024). Safety of pulsed field ablation in more than 17,000 patients with atrial fibrillation in the MANIFEST-17K study. Nat Med.

[12] Heiden C., Bejinariu A.G., Kelm M., Spieker M., Rana O. (forthcoming 2025). Hemolysis after pulsed-field ablation in pulmonary vein isolation for atrial fibrillation: A prospective controlled trial. Heart Rhythm.

[13] La Fazia V.M., Mohanty S., Torlapati P.G. (2025). Hydration to prevent kidney injury after pulsed field ablation: importance of timing and fluids amount. JACC Clin Electrophysiol.

[14] Lakkireddy D., Katapadi A., Garg J. (2025). Nemesis-PFA: investigating collateral tissue injury associated with pulsed field ablation. JACC Clin Electrophysiol.

[15] Charitakis E., Walfridsson H., Alehagen U. (2016). Short-term influence of radiofrequency ablation on NT-proBNP, MR-proANP, copeptin, and MR-proADM in patients with atrial fibrillation: data from the observational Smurf study. J Am Heart Assoc.

